# *Trichomonas tenax* induces barrier defects and modulates the inflammatory cytotoxicity of gingival and pulmonary epithelial cells*

**DOI:** 10.1051/parasite/2023010

**Published:** 2023-03-27

**Authors:** Zih-Bin Hong, Yu-Ting Lai, Chun-Hsien Chen, Yi-Jen Chen, Chien-Chin Chen, Wei-Chen Lin

**Affiliations:** 1 Institute of Basic Medical Sciences, College of Medicine, National Cheng Kung University Tainan Taiwan; 2 Department of Chest Division, Internal Medicine, Ditmanson Medical Foundation Chiayi Christian Hospital Chiayi Taiwan; 3 Department of Parasitology, College of Medicine, National Cheng Kung University Tainan Taiwan; 4 Department of Microbiology and Immunology, College of Medicine, National Cheng Kung University Tainan Taiwan; 5 Department of Pathology, Ditmanson Medical Foundation Chia-Yi Christian Hospital Chiayi Taiwan; 6 Department of Cosmetic Science, Chia Nan University of Pharmacy and Science Tainan Taiwan; 7 Department of Biotechnology and Bioindustry Sciences, College of Bioscience and Biotechnology, National Cheng Kung University Tainan Taiwan; 8 Rong Hsing Research Center for Translational Medicine, National Chung Hsing University 402 Taichung Taiwan

**Keywords:** *Trichomonas tenax*, Cytotoxicity, Cell junctions, IL-6

## Abstract

*Background:* *Trichomonas tenax* is a single-cell flagellated anaerobic organism, commensal in the human oral cavity. Although a previous study indicated that *T. tenax* could cause cell damage and phagocytose host epithelial cells, its pathological effects on gum cells remain unknown. Furthermore, several case reports have detected *T. tenax* in several patients with empyema and/or pleural effusion, which may have been aspirated from the oral cavity. However, the cytotoxic effects and immune responses of alveolar cells are unknown. Therefore, we aimed to determine the cytotoxic and immune effects of *T. tenax* on gums and pulmonary cell lines. The cytopathic effect and lactate dehydrogenase (LDH) cytotoxicity assays were used to determine the level of cell damage in gum and lung epithelial cells. Western blot was used to determine the disruption of cell junctions. Finally, epithelial cell cytokines were measured using ELISA to elucidate the immune response to *T. tenax*. *Results*: We found that *T. tenax *induced a cytotoxic effect on gum epithelial cells by disrupting cell junctions; however, it hardly triggered cellular damage in alveolar A549 cells and mucoepidermoid NCI-H292 cells. Furthermore, *T. tenax *induced the production of IL-6 at a low multiplicity of infection (MOI) in gum, A549, and NCI-H292 cells. *Conclusions*: Our results suggest that *T. tenax* can trigger gingival cell cytotoxicity, disrupt cell junctions, and induce IL-6 production in gingival and pulmonary cell lines.

## Abstract (fr)

*Contexte :* *Trichomonas tenax* est un organisme anaérobie unicellulaire flagellé, commensal dans la cavité buccale humaine. Bien qu'une étude précédente ait indiqué que *T. tenax* pouvait endommager les cellules et phagocyter les cellules épithéliales de l'hôte, ses effets pathogènes sur les cellules gingivales restent inconnus. En outre, plusieurs rapports ont détecté *T. tenax* chez des patients présentant un empyème et/ou un épanchement pleural, qui peut avoir été aspiré de la cavité buccale. Cependant, les effets cytotoxiques et les réponses immunitaires des cellules alvéolaires sont inconnus. Par conséquent, nous avons cherché à déterminer les effets cytotoxiques et immunitaires de *T. tenax* sur des lignées cellulaires de gencives et de poumons. Les tests d'effet cytopathique et de cytotoxicité de la lactate déshydrogénase (LDH) ont été utilisés pour déterminer le niveau de dommages cellulaires dans les cellules. Le Western Blot a été utilisé pour déterminer la perturbation des jonctions cellulaires. Enfin, les cytokines des cellules épithéliales ont été mesurées par ELISA pour élucider la réponse immunitaire à *T. tenax*. *Résultats :* Nous avons constaté que *T. tenax* induisait un effet cytotoxique sur les cellules épithéliales gingivales en perturbant les jonctions cellulaires. Cependant, *T. tenax* a déclenché des dommages cellulaires seulement mineurs dans les cellules alvéolaires A549 et les cellules mucoépidermoïdes NCI-H292. De plus, *T. tenax* a induit la production d'IL-6 à une faible multiplicité d'infection (MOI) dans les cellules de gencives, A549 et NCI-H292. *Conclusions :* Nos résultats suggèrent que *T. tenax* peut déclencher la cytotoxicité des cellules gingivales, perturber les jonctions cellulaires et induire la production d'IL-6 dans les lignées cellulaires gingivales et pulmonaires.

## Introduction

*Trichomonas tenax* is a single-cell flagellated anaerobic organism. It is typically found in the human oral cavity and is transmitted via saliva droplets, drinking water, and contaminated food [14]. *Trichomonas tenax* is a known oral commensal protozoa and is distributed widely among 4–53% of the population [11]. However, studies have indicated that the detection rate is higher in patients with poor oral hygiene, and its presence was highly correlated with periodontal disease in a cohort study [2, 11]. Based on a systematic review and meta‑analysis, *T. tenax* was shown to have a relationship with candidiasis, gingivitis and periodontitis in pooled prevalence [8]. In addition, a previous study showed a relationship between the severity of periodontitis and the detection of *T. tenax* in dental plaque [16]. However, the pathogenicity and virulence of *T. tenax* are still unknown. Therefore, it is crucial to determine the interaction between *T. tenax *and gum tissue.

In a previous study, transmission electron microscopy (TEM) revealed that *T. tenax* could attach to mammalian cells and form aggregates that coated the surface of epithelial cells until disruption of the monolayer [18]. Furthermore, cytotoxicity and phagocytosis were observed in Madin-Darby canine kidney (MDCK) and HeLa cells during incubation with *T. tenax*. Similar results were observed in the presence of *T. vaginalis *and host cell incubation [18]. Although *T. tenax* induces cell damage, the exact oral pathology underlying this interaction between *T. tenax* and gum cells remains unclear. Previous studies have shown that most cases of bacterial pneumonia result from oral and/or pharyngeal flora that are obtained primarily by aspiration and inhalation, especially in patients with periodontal disease or poor oral hygiene [15, 19, 20]. Therefore, the presence of oral pathogens could increase the risk of pneumonia in elderly or immunodeficient patients following aspiration or inhalation. *Trichomonas tenax* is the most frequent species of trichomonads that causes pulmonary trichomoniasis accompanied by pyopneumothorax and empyema [26, 27]. Sequencing analysis showed that almost 30% of bronchoalveolar lavage fluid samples from 77 patients in the ICU contained *T. tenax*. Additionally, among these confirmed cases, 17 patients were also associated with acute respiratory distress syndrome (ARDS) [6]. Although many clinical cases of *T. tenax* in the respiratory tract are associated with aspiration pneumonia, the process of pulmonary immunity and pathology during *T. tenax* invasion remains largely unknown. A previous study showed that incubation with the lysate of *T. tenax* directly stimulated the production of interleukin-8 in THP-1 cells [9]. However, the link between *T. tenax *and lung epithelial cells remains unknown. Therefore, it is necessary to determine the interaction between *T. tenax* and pulmonary epithelial cells to identify the role of *T. tenax* in pulmonary immunity and pathology.

In this study, we analyzed the integration of the epithelial barrier and polarity following the incubation of human gingival epithelial Smulow-Glickman (S-G) cells with *T. tenax. *Our results revealed that host cells lost tight junctions and showed reduced viability during coculture with *T. tenax, *as shown by western blot and cytopathic assays. Additionally, we demonstrated the production of proinflammatory cytokines by pulmonary epithelial cells after coculture with* T. tenax*. Our findings indicated that IL-6 was induced in the absence of cell damage, which indicates a role of lung immunity in pulmonary trichomoniasis.

## Materials and methods

### Host cells and Trichomonas tenax

Human gingival epithelial Smulow-Glickman (S-G) cells (provided by Dr. Jenn-Wei Chen, Department of Microbiology and Immunology, College of Medicine, National Cheng Kung University, Tainan, Taiwan), A549 lung cancer cells (ATCC_CCL-185), and NCI-H292 mucoepidermoid pulmonary cells (ATCC_ CRL-1848) were cultured in Dulbecco’s modified Eagle’s medium (DMEM, Gibco^TM^, Thermo Fisher Scientific, Waltham, MA, USA) or Roswell Park Memorial Institute (RPMI 1640 Medium, Gibco^TM^, Thermo Fisher Scientific) supplemented with 10% heat-inactivated fetal bovine serum (FBS, Gibco^TM^, Thermo Fisher Scientific) and 100 units/mL penicillin–streptomycin (Thermo Fisher Scientific) and were maintained in a humidified atmosphere with 5.0% CO_2_ at 37 °C. *Trichomonas tenax *(ATCC_30207) was cultured in 20 mL of YI-S medium (yeast extract, iron-serum) supplemented with 10% heat-inactivated horse serum (Gibco^TM^, Thermo Fisher Scientific) in culture flasks under anaerobic conditions at 37 °C and was harvested in the logarithmic phase after incubation for 24 h.

### Host cell–T. tenax coculture conditions

S-G, A549, and NCI-H292 cells were harvested and seeded in 6- or 24-well plates overnight in 5.0% CO_2_ at 37 °C to form a monolayer. For coculture conditions, *T. tenax* was harvested from the culture medium. Next, host cells and *T. tenax* at MOIs of 1, 2, 4, or 8 were cocultured in DMEM or RPMI medium without FBS and antibiotics for 24 h. The coculture medium was collected for immunoassays or cell-mediated cytotoxicity assays, and the remaining cells were used for cytopathic effect assays or observed with a Cell^R^ microscope (Olympus CellR, Tokyo, Japan).

### Cytopathic effect (CPE) assay

After host cells and *T. tenax* were cocultured for 24 h, the supernatant was removed, and the remaining cells were washed gently with 1 mL of phosphate-buffered saline (PBS). The cells were fixed with methanol and acetic acid at a ratio of 3:1 for 30 min. After air drying, Giemsa buffer (Merck, Darmstadt, Germany) was mixed with PBS buffer at a ratio of 9:1, added to 200 μL and incubated for 30 min. The wells were then rinsed with ddH_2_O and allowed to air dry. The results were observed using a Cell^R^ microscope (Olympus CellR, Tokyo, Japan) and quantified using ImageJ software.

### Lactate dehydrogenase (LDH) cytotoxicity assay

The coculture medium was examined by a CytoTox 96^®^ Non-Radioactive Cytotoxicity Assay (Promega, Madison, WI, USA). The medium was centrifuged at 250 ×g for 4 min to obtain a supernatant that was mixed with the CytoTox 96 Reagent. The mixture was protected from light for 30 min until the addition of the stop solution. The absorbance was recorded at 490 nm using a microplate spectrophotometer (Thermo Fisher Scientific) and analyzed using GraphPad Prism 7 (GraphPad Software, La Jolla, CA, USA).

### Western blot analysis

Protein samples were extracted using RIPA buffer (Thermo Fisher Scientific) and separated using 12% sodium dodecyl sulfate–polyacrylamide gel electrophoresis (SDS–PAGE, T-Pro, Taipei City, Taiwan) for western blot analysis. The proteins were then transferred to polyvinylidene difluoride (PVDF) membranes (Merck Millipore, Darmstadt, Germany). The membranes were blocked in 5% nonfat dry milk in 0.5% PBS-T. Immune complexes were formed by incubating the proteins with primary antibodies overnight at 4 °C (anti-ZO-1, Abcam, Waltham, MA, USA; anti-E-cadherin, Elabscience, Houston, TX, USA; anti-human desmoplakin 1&2, EMD Millipore, Burlington, MA, USA; anti-JAM-A, Invitrogen, Waltham, MA, USA; anti-β-catenin, EMD Millipore, USA; anti-connexin-43, Sigma-Aldrich, Saint Louis, MO, USA; anti-β-actin, EMD Millipore, USA). Then, the membranes were washed and incubated for 1 h with goat anti-mouse or rabbit HRP-tagged secondary antibodies (1:5000; Leadgen, Tainan, Taiwan). Immunoreactive protein bands were subsequently detected using a luminescence/fluorescence imaging system (GE Healthcare).

### Enzyme-linked immunosorbent assay (ELISA)

The coculture supernatants were used to measure human IL-6, IL-1β, and TNFα levels using ELISA kits (BioLegend, San Diego, CA, USA). The samples were centrifuged at 250 ×*g *for 4 min, and the supernatant was tested by ELISA, according to the manufacturer’s instructions. The absorbance was recorded at 450 nm and 570 nm using a microplate spectrophotometer (Thermo Fisher Scientific) and graphed using GraphPad Prism 7 software.

### Statistical analysis

The data are presented as the mean ± standard deviation (SD). All comparisons were conducted using an unpaired two-tailed Student’s *t *test. Statistical significance was set at *p < *0.05. The data were analyzed statistically using GraphPad software (version 7.0).

## Results

**Figure 1:**
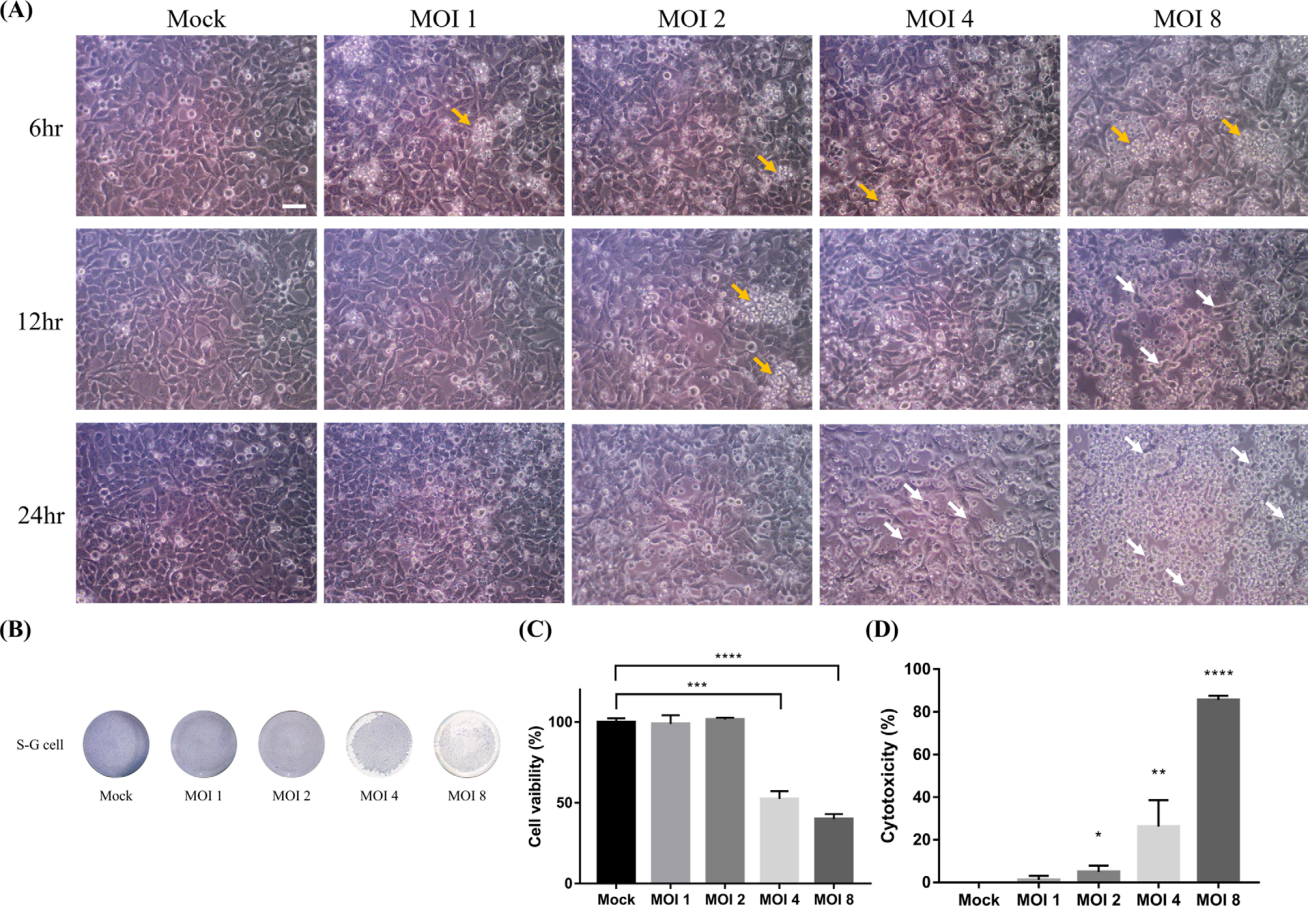
Induction of cell cytotoxicity during *Trichomonas tenax* incubation with S-G cells for 24 h. (A) The cell viability of S-G cells incubated with *T. tenax* at different MOIs (1, 2, 4, 8) for 6, 12, and 24 h was assessed using a microscope (Yellow arrow: aggregated *T. tenax*, white arrow: round S-G cell shape, Scale Bar: 50 μm). (B) The CPE of S-G cells co-incubated with *T. tenax* was observed by staining with Giemsa buffer in 24-well plated. (C) Stained cells were calculated by ImageJ. (D) Lactate dehydrogenase (LDH) cytotoxicity assay was performed on S-G cells after coincubation with *T. tenax* at different MOIs for 24 h (*p ≤ 0.05, **p ≤ 0.01, ***p ≤ 0.001, ****p ≤ 0.0001, MOI: multiplicity of infection).

*Trichomonas tenax* could disrupt the monolayers of MDCK and HeLa cells and induce host cell damage. Therefore, in this study, we investigated the viability of S-G cells during incubation with *T. tenax.* The coculture conditions were 6, 12, and 24 h and MOIs of 1, 2, 4, and 8. The cytotoxicity of S-G cells gradually increased by incubation with *T. tenax* at MOIs of 4 and 8 for 12 to 24 h (Fig. 1A). As indicated by the white arrow in Figure 1A, shrinkage and rounding of S-G cells were observed at MOI 4 and MOI 8 after incubation for 24 h and 12 h, respectively. It was further observed that most of the cells detached from the bottom of the well after incubation with an MOI of 8 for 24 h (Fig. 1A). Furthermore, approximately 50% CPE was observed at an MOI of 4 and 8 in S-G cells after 24 h of incubation by staining with Giemsa buffer (Figs. 1B and 1C). *Trichomonas tenax* further induced the gradual release of LDH from S-G cells at MOIs of 2–8, which is consistent with the CPE results (Fig. 1D). In addition, *T. tenax* had little effect on LDH release in this coculture system. Therefore, the results of the present study indicate that *T. tenax* at MOIs of 4 and 8 triggers cell damage in S-G cells.

**Figure 2:**
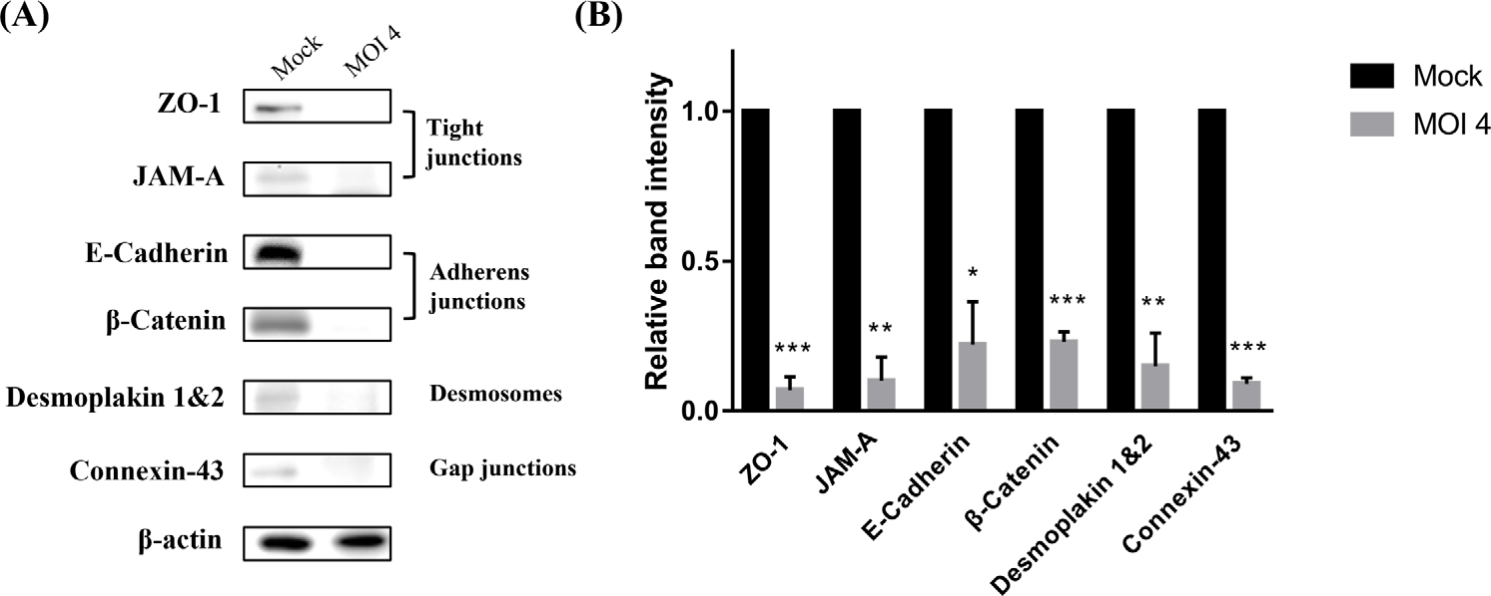
Reduction in cell junction protein expression in S-G cells caused by *Trichomonas tenax*. (A) Western blotting results of cell junctions (ZO-1, JAM-A, E-cadherin, β-catenin, desmoplakin 1 & 2, and connexin) expression levels of S-G cells stimulated by *T. tenax* at MOI 4 for 24 h. (B) Relative quantification of western blotting results was performed with ImageJ (*p ≤ 0.05, **p ≤ 0.01, ***p ≤ 0.001, MOI: multiplicity of infection).

**Figure 3:**
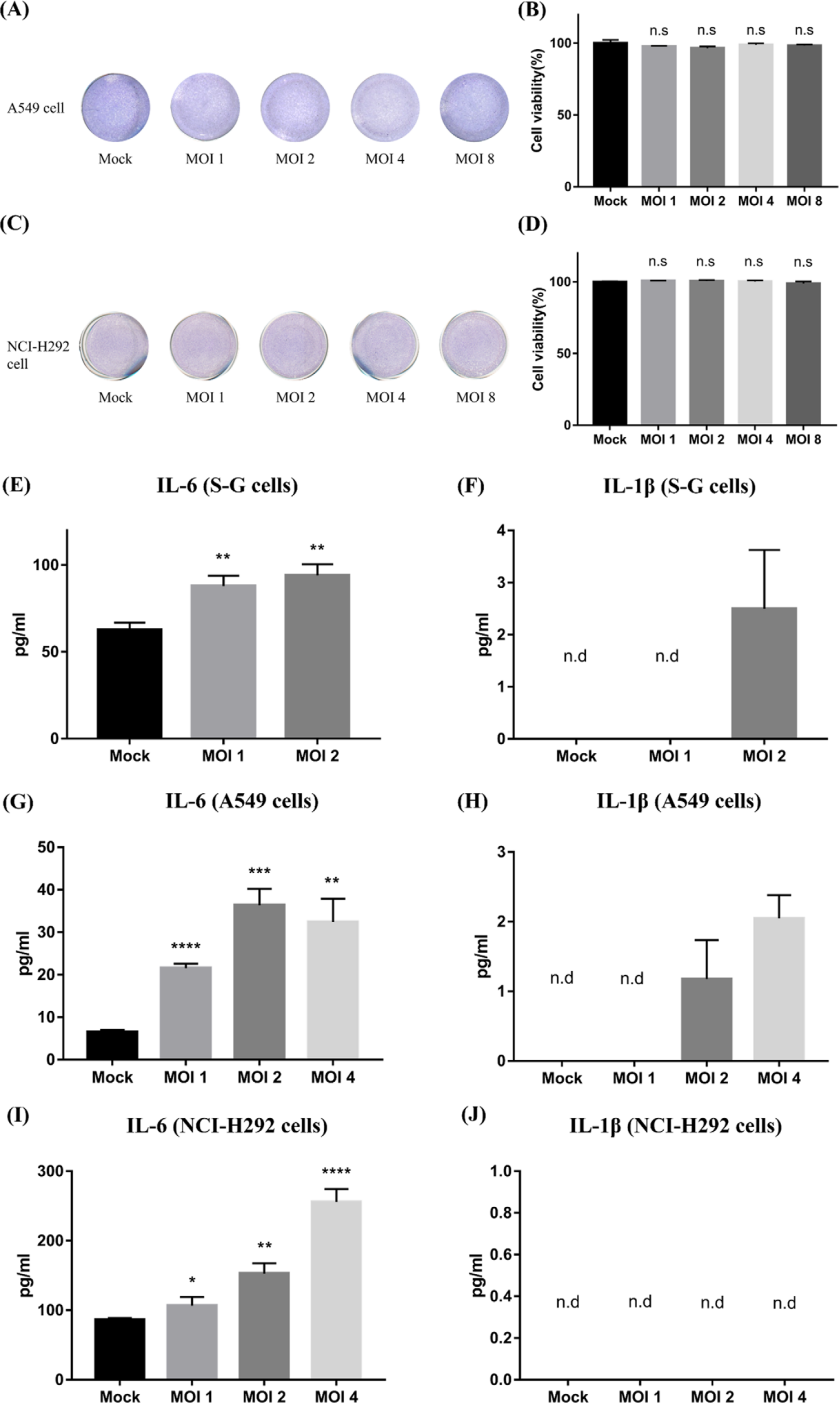
Cytopathic effect and cytokine production of S-G, A549, and NCI-H292 cells after stimulation by *Trichomonas tenax*. A549 (A, B) and NCI-H292 cells (C, D) were co-incubated with *T. tenax* for 24 h, the attached cells were then stained with Giemsa buffer and the percentage of stained cells was calculated by ImageJ. S-G (E, F), A549 (G, H) and NCI-H292 (I, J) cells were treated with *T. tenax* at different MOIs for 24 h, and the concentration of IL-6 and IL-1β were detected by ELISA (n.d: not detection, n.s: not significant, **p ≤ 0.01, ***p ≤ 0.001, ****p ≤ 0.0001, MOI: multiplicity of infection).

Due to the cytopathic effect of *T. tenax*, we examined whether cell junctions, which play important roles in the regulation of oral barrier function, were destroyed by *T. tenax.* The integration of cell junctions such as tight junctions, adherens junctions, desmosomes, and gap junctions was analyzed. The western blot results showed that *T. tenax* at an MOI of 4 dramatically decreased the expression of ZO-1, JAM-A, E-cadherin, β-catenin, desmoplakin 1 and 2, and connexin-43 in S-G cells (Fig. 2). In addition to the destruction of the cell barrier, the release of cytokines is an important factor in the onset of oral infection for disease progression. Our data showed that IL-6 was significantly higher in the MOI 1 and 2 groups than in the mock group after S-G cells were cocultured with *T. tenax *for 24 h (Fig. 3E). However, IL-1β was hardly detected in any of the groups after the incubation of S-G cells with *T. tenax* (Fig. 3F). TNFα, which was not shown in our data, was also undetectable by ELISA, regardless of the MOI. Taken together, these results indicate that* T. tenax* could induce the release of IL-6 from S-G cells, whereas IL-1β and TNFα were hardly induced in gum epithelial cells. These data reveal that *T. tenax* not only induces cytopathology but also promotes the release of proinflammatory cytokines from gum epithelial cells in the human oral cavity.

A previous clinical case report showed that patients who had pulmonary trichomoniasis exhibited sepsis-like syndrome and bronchospasm. In addition, some patients with late ARDS and empyema were shown to have *T. tenax* in their bronchoalveolar lavage. In addition to the damage induced by *T. tenax* in S-G cells, we further evaluated the cytopathic effect and cytokine production in A549 and NCI-H292 epithelial cells in the presence of *T. tenax*. There was no significant difference in the cytopathic effect on either cell line in response to MOIs of 1, 2, 4, and 8 (Figs. 3A, 3B, 3D). In addition, cell junctions were tightly bound under all conditions. Next, we investigated whether* T. tenax* induced cytokine production in A549 and NCI-H292 cells. Our data showed that IL-6 production was induced in both cell types after *T. tenax* stimulation for 24 h (Figs. 3G, 3I). However, IL-1β was barely induced in any of the groups of A549 and NCI-H292 cells after incubation with *T. tenax* (Figs. 3H, 3J). TNFα was below the detection value in A549 and NCI-H292 cells. These data suggest that *T. tenax* has little effect on cytopathology, but can induce IL-6 secretion in pulmonary cell lines. This finding indicates that proinflammatory cytokines are released and induce downstream immunity following the invasion of *T. tenax* through the pulmonary barrier.

## Discussion

**Figure 4:**
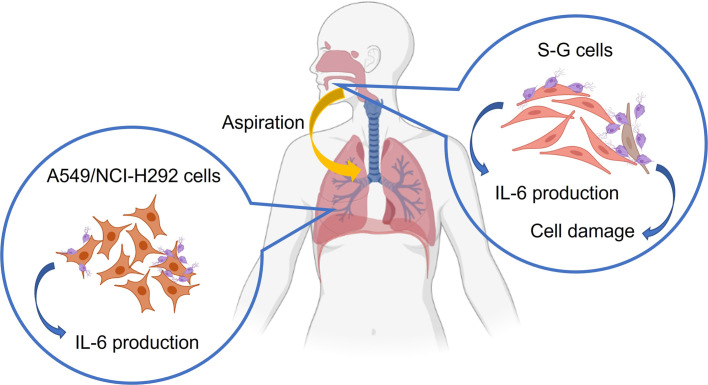
The overall effect of pathology and immune response that *Trichomonas tenax* induced on gum S-G cells and alveolar A549 cells. S-G cells was damaged at high MOI, while the production of IL-6 was induced at low MOI. A549 cells induced IL-6 at both low and high MOI. The figure was created with https://www.biorender.com/.

In this study, we investigated the pathological and immunological effects of *T. tenax* on gum S-G cells and alveolar A549 cells. Our results showed that *T. tenax* triggered cytotoxic effects on S-G cells with increasing MOI levels. We further found that cell junctions, including tight junctions, adherens junctions, desmosomes, and gap junctions, were compromised when epithelial barriers were damaged (Fig. 4). These data correspond with the cytopathic effect of *T. tenax* on MDCK and HeLa cells reported in a previous study [18]. In addition, the data suggested that *T. tenax* could induce the immune response in the epithelium and was associated with periodontitis. Although several studies have previously reported the symptoms of pulmonary trichomoniasis, the underlying pathological mechanism remains unknown [11, 13, 14]. Our research showed that *T. tenax* did not damage A549 and NCI-H292 cells and did not induce the disruption of cell junctions (Fig. 4). However, cytokines were produced after incubation with *T. tenax*, which may be linked to the related immune response involving neutrophil recruitment and empyema in the pulmonary system [12, 14].

Several studies have shown that several proteases isolated from clinical samples of *T. tenax *were identified by protein profiling [7, 21]. These proteases were also shown to have proteolytic activity, which may promote their pathogenicity in oral and pulmonary invasion [7, 21]. Cysteine proteinases (CPs) which are the most abundant soluble secretory proteins of *Trichomonas vaginalis* play important roles in cytotoxicity, adhesion, and apoptosis [17, 24]. Monolayer HeLa cells were treated with CP65 of *T. vaginalis,* and cellular pathology was induced [1]. Furthermore, it was shown that CP30 of *T. vaginalis* could trigger apoptosis in human vaginal epithelial cells (HVECs) [22]. Another study indicated that incubation with *T. vaginali*s resulted in the disruption of junctional complex proteins in Caco-2 cells, while the same effect was observed in IPEC-J2 cells incubated with *T. foetus* [4, 25]. Therefore, the function and activity of the cysteine proteinases of *T. tenax* need to be verified.

When challenged with *Trichomonas vaginalis,* the production of IL-6 in RWPE-1 cells was increased [10]. These results are similar to our data showing that IL-6 was produced by S-G, A549, and NCI-H292 cells that were incubated with *T. tenax*. A previous study showed that several oral pathogenic bacteria could induce the release of inflammatory cytokines from primary human gingival epithelial cells [23]. Interestingly, one study revealed that *Aggregatibacter actinomycetemcomitans,* which is associated with periodontal inflammation, could induce a low IL-1β response and high IL-6 response [23]. Therefore, our results might indicate a unique cytokine hallmark during the incubation of gum cells with *T. tenax*. Regarding the pulmonary system, several case reports have shown that patients infected with* T. tenax* had empyema and leukocytosis [12]. Furthermore, it has been reported that pleural effusion and high leukocyte counts were highly correlated with IL-6 concentrations [5]. In allergies, the cysteine protease produced by house dust mites (HDM), Der p 1, can cleave several cellular junctions to penetrate the subepithelial tissue and induce an immune response [3]. Similar to the results of our study, Der p 1 can trigger the production of IL-6 in the airway epithelium [3]. Several studies have also revealed that lysates from clinically isolated *T. tenax* exert proteolytic activity and contain several cysteine proteases ranging in size from 14 to 66 kDa [7, 21]. These proteases may play roles in the induction of cell damage and cytokine production in these epithelial cells. Thus, more detailed experiments are required to determine the correlation between pulmonary immunology and *T. tenax* infection.

## Conclusions

Our data have shown that* T. tenax* can induce a cytopathic effect on gingival cells. In addition, several cell–cell junctions were disrupted by an MOI of 4. In contrast, pulmonary epithelial cells were hardly damaged after incubation. Proinflammatory cytokines, especially IL-6, were released from gum and pulmonary epithelial cells after coculture with *T. tenax*. Therefore, our data indicate that *T. tenax* damages different cells to different degrees, but all contribute to the induction of a similar inflammatory response.

## Competing interests

The authors declare that they have no competing interests.
